# Overcoming MDSC-Mediated Immunosuppression in Hepatocellular Carcinoma: From Mechanisms to Novel Immunotherapeutic Approaches

**DOI:** 10.3390/cancers18060980

**Published:** 2026-03-18

**Authors:** Yangzhi Ou, Huaxiu Wei, Chunxiu Peng, Jin Li, Ke Wei, Chenjie Zhan, Zhiyong Zhang

**Affiliations:** 1 State Key Laboratory of Targeting Oncology, Guangxi Medical University, Nanning 530021, China; 202321617@sr.gxmu.edu.cn (Y.O.); 202321625@sr.gxmu.edu.cn (H.W.); 202410267@sr.gxmu.edu.cn (C.P.); 202421681@sr.gxmu.edu.cn (J.L.); 202221579@sr.gxmu.edu.cn (K.W.); 202221564@sr.gxmu.edu.cn (C.Z.); 2 National Center for International Research of Bio-Targeting Theranostics, Guangxi Medical University, Nanning 530021, China; 3 Guangxi Key Laboratory of Bio-Targeting Theranostics, Guangxi Medical University, Nanning 530021, China; 4 Collaborative Innovation Center for Targeting Tumor Diagnosis and Therapy, Guangxi Medical University, Nanning 530021, China; 5 Guangxi Talent Highland of Major New Drugs Innovation and Development, Guangxi Medical University, Nanning 530021, China; 6 Targeting Theranostics Research Center of Guangxi Higher Education, Guangxi Medical University, Nanning 530021, China; 7 Department of Surgery, Robert-Wood-Johnson Medical School University Hospital, Rutgers University, New Brunswick, NJ 08901, USA

**Keywords:** hepatocellular carcinoma, myeloid-derived suppressor cells, immunosuppression, tumor microenvironment, JAK–STAT3 signaling, metabolic reprogramming, epigenetic remodeling, immune checkpoint blockade, combinatorial therapy, microbiome interventions

## Abstract

**Background**: Myeloid-derived suppressor cells (MDSCs) drive immunosuppression in the hepatocellular carcinoma (HCC) tumor microenvironment (TME), contributing to immune checkpoint blockade (ICB) resistance. This review explores underlying mechanisms and therapeutic strategies. **Methods**: We synthesize the recent literature on MDSC biology in HCC, focusing on signaling pathways, metabolic/epigenetic reprogramming, and novel interventions, including AI-driven analyses. **Results**: Key mechanisms include JAK–STAT3 activation for MDSC expansion, CXCL12-CXCR4 for recruitment, enhanced glycolysis/lipid metabolism for suppressive function, and epigenetic changes sustaining immunosuppression. Therapeutic approaches encompass inhibitors, differentiation promoters, metabolic modulators, transcriptional reprogramming, microbiome modulation, and combinations with ICB/locoregional therapies or standard chemoimmunotherapy, yielding improved outcomes in trials. **Conclusions**: Targeting MDSC redundancies via multi-modal strategies offers a roadmap for overcoming resistance, with AI enhancing biomarker-guided precision immunotherapy in HCC.

## Simple Summary

Myeloid-derived suppressor cells (MDSCs) play a central role in promoting immune evasion and resistance to immune checkpoint blockade (ICB) therapies in hepatocellular carcinoma (HCC), a leading cause of cancer-related deaths worldwide. This review elucidates key mechanisms driving MDSC-mediated immunosuppression, including interconnected signaling pathways like JAK–STAT3 and CXCL12-CXCR4, metabolic reprogramming such as enhanced glycolysis and lipid metabolism, and epigenetic modifications that sustain tumor tolerance. Emerging therapeutic strategies, including STAT3 inhibitors, metabolic modulators (e.g., tadalafil and SQLE blockers), microbiome interventions via fecal microbiota transplantation or probiotics, and combinations with ICB or locoregional therapies like transarterial chemoembolization, demonstrate promising efficacy in preclinical and clinical studies by depleting MDSCs, reprogramming the tumor microenvironment, and enhancing antitumor immunity. These insights position MDSCs as pivotal targets for precision immunotherapy, with AI-driven multi-omics integration facilitating biomarker discovery and personalized regimens to improve response rates and patient outcomes in HCC.

## 1. Introduction

Hepatocellular carcinoma (HCC) stands as one of the top causes of cancer deaths globally, driven by its aggressive nature, frequent recurrence, and limited treatment options—all exacerbated by ongoing liver inflammation from etiologies such as viral hepatitis, non-alcoholic fatty liver disease (NAFLD), or heavy alcohol use, which fosters a naturally tolerogenic liver environment prone to tumor immune evasion [1]. Myeloid-derived suppressor cells (MDSCs) comprise a heterogeneous group of immature myeloid cells that expand during chronic inflammation or cancer. They originate primarily from bone marrow common myeloid progenitors (CMPs) and immature myeloid cells (IMCs), with their primary function being to suppress antitumor immune responses by inhibiting T-cell and natural killer (NK)-cell activity [2]. Within the tumor microenvironment (TME), these cells remain in an immature state, bypassing differentiation into mature neutrophils, macrophages, or dendritic cells, leading to their accumulation and enhanced immunosuppressive capacity [3]. In HCC, elevated MDSC levels are observed in both peripheral blood and tumor tissues, correlating with advanced disease stages, poorer prognosis, and treatment resistance. For patients with chronic viral hepatitis or NAFLD, these increased MDSC counts serve as prognostic indicators of adverse outcomes [4]. MDSCs achieve immune suppression through mechanisms such as cytokine release (e.g., IL-10 and TGF-β) that inhibit T-cell proliferation, metabolic alterations depleting arginine or elevating reactive oxygen species (ROS) to impair T-cell function, and interactions with other suppressors like regulatory T cells (Tregs) and tumor-associated macrophages (TAMs) to form reinforcing loops [5]. The liver’s inherent tolerance, amplified by chronic inflammation, provides an ideal niche for MDSC proliferation and activation. Tumor-derived chemokines, including CCL2 and CXCL12, recruit MDSCs, intensifying the immunosuppressive TME [6]. This effect extends systemically, inducing widespread immune suppression [7]. Although ICB has revolutionized treatment in other cancers, its efficacy in HCC is limited by MDSCs, which hinder T-cell and NK-cell responses and promote resistance [8]. MDSC recruitment and activation involve pathways such as JAK–STAT3 for expansion, CXCL12–CXCR4 for migration, metabolic shifts (e.g., enhanced glycolysis and lipid oxidation), and epigenetic modifications like DNA methylation or histone alterations [9,10]. These mechanisms highlight potential MDSC-targeting strategies, including STAT3 inhibitors, metabolic modulators, or microbiome interventions, particularly when combined with ICB to enhance efficacy and overcome resistance [11]. Recently, artificial intelligence (AI) has emerged as a promising tool for dissecting the complex tumor microenvironment (TME) in hepatocellular carcinoma (HCC), particularly in relation to MDSC-mediated immunosuppression [12,13]. Recent multi-omics integration combined with machine learning has successfully identified distinct HCC molecular subtypes with differential immunosuppressive features. For instance, delineated two consensus subtypes (CS1 and CS2) using multi-omics data, where the CS2 subtype exhibits higher mutation burden, enriched MDSC and CAF infiltration, increased T-cell dysfunction/exclusion scores, and poorer immunotherapy response, highlighting AI’s potential for patient stratification toward MDSC-modulating combinatorial regimens [14]. Similarly, applied machine learning to define disulfidptosis-related subtypes in HCC, revealing that high-risk subtypes display elevated immunosuppressive cell infiltration (including MDSCs) and reduced immunotherapy efficacy, suggesting disulfidptosis-linked metabolic vulnerabilities as adjunct targets to overcome MDSC-driven resistance [15]. Furthermore, integrated machine learning approaches have identified glutamine metabolism biomarkers such as SLC1A5, which predict immunotherapy response and correlate with immunosuppressive TME features in HCC [16]. These advances, supported by broader applications of AI and machine learning in HCC management [17], underscore AI/multi-omics as valuable tools for biomarker discovery and precision targeting of MDSC redundancies, despite ongoing challenges in model interpretability and clinical validation.

## 2. Origins, Heterogeneity, and Dynamics of MDSCs in Hepatocellular Carcinoma

Myeloid-derived suppressor cells (MDSCs) represent a heterogeneous population of immature myeloid cells that expand aberrantly during chronic inflammation and tumorigenesis, exerting pivotal immunosuppressive functions within the hepatocellular carcinoma (HCC) tumor microenvironment (TME) [18]. Derived from bone marrow common myeloid progenitors (CMPs) and immature myeloid cells (IMCs), pathological conditions arrest their differentiation into mature neutrophils, macrophages, or dendritic cells, instead conferring potent immunosuppressive activity [3]. Human MDSCs are classified into monocytic (M-MDSC; CD11b^+^CD33^+^CD14^+^HLA-DR^low/−CD15^−^), polymorphonuclear MDSC (PMN-MDSC, also known as granulocytic MDSC; CD11b^+^CD33^+^CD14^−^CD15^+^ or CD66b^+^HLA-DR^low/−), early-stage (eMDSC; Lin^−^[CD3/CD14/CD15/CD19/CD56] HLA-DR^−^CD33^+^CD11b^+^/low), and emerging fibrocytic subsets (CD11b^+^CD33^+^HLA-DR^−^ with fibrocyte markers such as Collagen I^+^ and CD34^+^) [19,20].

In HCC, these subsets exhibit distinct recruitment patterns, suppressive mechanisms, and prognostic implications (summarized in Table 1). M-MDSCs predominantly mediate immunosuppression through elevated arginase-1 (Arg-1), inducible nitric oxide synthase (iNOS), nitric oxide (NO), transforming growth factor-β (TGF-β), interleukin-10 (IL-10), and prostaglandin E2 (PGE2) secretion, as well as induction of regulatory T cell (Treg) differentiation; they can further differentiate into tumor-associated macrophages (TAMs). PMN-MDSCs, the predominant subtype (comprising 70–90% of total MDSCs in peripheral blood), primarily rely on reactive oxygen species (ROS), peroxynitrite (PNT), Arg-1, cationic amino acid transporter 2B (CAT2B), and S100A8/A9 to impair T-cell activity, correlating with advanced tumor stage, poor prognosis, and chemotherapy resistance [3]. eMDSCs, enriched in the peripheral blood of cancer patients and scarce in healthy individuals, exhibit mixed suppressive mechanisms (Arg-1, ROS, TGF-β) depending on maturation stage and are elevated in early-stage malignancies and autoimmune conditions [19]. Fibrocytic MDSCs, potentially arising from M-MDSCs under chronic inflammatory cues, localize to tumor stroma and sites of persistent inflammation, promoting potent TGF-β production, extracellular matrix remodeling, fibrosis, and angiogenesis; they are particularly prominent in fibrotic tumors, including those associated with HCC [20,21]. MDSC accumulation and activation are central to immune evasion throughout HCC progression. MDSC frequencies are markedly elevated in peripheral blood, tumor tissues, lymph nodes, and stroma of HCC patients, correlating with advanced disease stage, unfavorable prognosis, and therapeutic resistance [22]. Beyond local TME accumulation, MDSCs induce systemic immunosuppression, with MDSC-driven T-cell exhaustion serving as a key mechanism of immune checkpoint blockade (ICB) resistance [23,24]. The liver’s intrinsic tolerogenic properties, coupled with chronic inflammation from underlying etiologies (e.g., viral hepatitis and non-alcoholic fatty liver disease [NAFLD]), foster MDSC expansion and activation, including fibrocytic subsets that exacerbate fibrosis-driven HCC [21,25,26,27]. Tumor cells upregulate inhibitory ligands (e.g., PD-L1) and secrete chemokines (e.g., CCL2, CXCL12) to recruit MDSCs, thereby intensifying TME immunosuppression [20,28,29]. The dynamic evolution and heterogeneity of MDSCs underscore their complexity as therapeutic targets in HCC immunotherapy (summarized in Table 1) [30].

Summary of key phenotypic markers (human and mouse), primary suppressive mediators, tissue distribution, and prognostic/clinical associations of polymorphonuclear (PMN-MDSC), monocytic (M-MDSC), early-stage (eMDSC), and fibrocytic (F-MDSC) subsets in HCC.

### 2.1. The JAK–STAT3 Axis: A Master Regulator of MDSC Expansion and Immunosuppressive Potency

**Figure 1:**
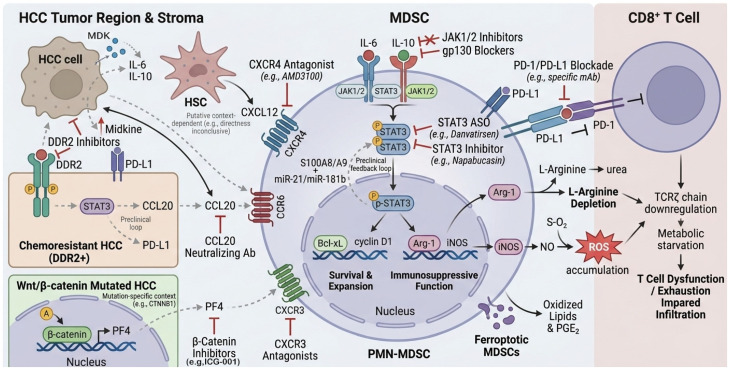
Molecular mechanisms of MDSC-mediated immunosuppression in HCC.

The Janus kinase–signal transducer and activator of transcription 3 (JAK–STAT3) axis is widely recognized as the central hub governing MDSC biology. In the HCC TME, persistent stimulation by proinflammatory cytokines—most notably IL-6 and IL-10—sustains STAT3 activation [32,33]. Activated STAT3 translocates to the nucleus, where it upregulates anti-apoptotic (e.g., Bcl-xL) and pro-proliferative (e.g., cyclin D1) genes, thereby promoting MDSC expansion and survival. Concurrently, STAT3 induces expression of arginase-1 (Arg-1) and inducible nitric oxide synthase (iNOS), leading to L-arginine depletion, reactive oxygen species (ROS) accumulation, downregulation of the T-cell receptor ζ-chain, and profound T-cell dysfunction [32] (see Figure 1).

Schematic representation of the major signaling pathways and feedback loops driving MDSC recruitment, expansion, and suppressive function in the hepatocellular carcinoma (HCC) tumor microenvironment, including the JAK–STAT3 axis, CXCL12/CXCR4 axis, DDR2–STAT3–CCL20 loop, and β-catenin–PF4–CXCR3 axis. Gray dashed lines indicate putative or context-dependent links primarily supported by preclinical evidence (see Section 2.1, Section 2.2, Section 2.3, Section 2.4 and Section 2.5).

In the setting of chronic viral hepatitis or chronic inflammation, sustained IL-6–gp130 signaling reinforces a positive feedback loop involving the S100A8/A9–STAT3–C/EBPβ complex and miR-21/miR-181b, further entrenching the immunosuppressive MDSC phenotype [34,35] (see Figure 1). Meta-analyses confirm that elevated phosphorylated STAT3 (p-STAT3) expression in tumor tissues independently predicts poorer overall survival in HCC patients, with pooled hazard ratios of 1.7 (95% CI 1.4–2.1) for 3-year OS and 1.9 (95% CI 1.5–2.4) for disease-free survival, based on meta-analysis of approximately 1000 patients (primarily Asian cohorts) [36].

Therapeutically, the STAT3 antisense oligonucleotide danvatirsen reduced STAT3 mRNA levels and achieved a 26% objective response rate in advanced solid tumors (Phase Ib/II trial, NCT03421353), accompanied by significant peripheral MDSC depletion; an HCC expansion cohort is ongoing [Phase Ib/II] [31]. The small-molecule STAT3 inhibitor napabucasin (BBI-608) disrupts STAT3-driven fatty acid oxidation in preclinical HCC models, alleviating TAM/MDSC-mediated T-cell exhaustion. Nano-formulated napabucasin extended survival by approximately 30% in preclinical HCC models (*N* = mouse groups of 8–10, comparator: vehicle control) and synergized with anti-PD-1 in murine models [37,38]. Given compensatory activation of upstream kinases (JAK, SRC), combined JAK1/2 or gp130 inhibition is under active investigation [33].

### 2.2. Midkine-Driven MDSC Recruitment via the CXCL12/CXCR4 Axis

Midkine (MDK) is significantly overexpressed in HCC tissues and sera, reshaping the tumor secretome to promote massive MDSC infiltration and blunt PD-1/PD-L1 blockade efficacy [39,40]. In orthotopic HCC models, MDK-overexpressing cells upregulate IL-10/STAT3 signaling, enhancing the suppressive capacity of CD11b^+^Gr-1^+^ MDSCs; conversely, MDK knockdown reverses this phenotype and restores anti-PD-1 sensitivity [39]. Although direct induction of CXCL12 by MDK remains inconclusive, the SDF-1α (CXCL12)/CXCR4 axis is a well-established chemokine pathway for MDSC trafficking to the liver, with spatial overlap observed in MDK-high regions [40] (see Figure 1).

CXCR4^+^ MDSCs are enriched in anti-PD-1-resistant HCC models. CXCR4 antagonists such as AMD3100 markedly reduced MDSC infiltration and reinvigorated CD8^+^ T-cell function in preclinical orthotopic HCC models [Preclinical] [41,42]. Activated hepatic stellate cells constitutively secrete CXCL12, establishing a CXCR4-dependent immunosuppressive axis [20]. Clinically, adding AMD3100 to sorafenib plus anti-PD-1 significantly attenuates MDSC infiltration and enhances tumor growth inhibition [41]. CXCR4-targeted CAR-T cells that disrupt the CXCL12 gradient are also being explored in humanized HCC models [43]. Blocking recruitment rather than directly ablating MDSCs offers a safer profile with reduced systemic toxicity [44]. Moreover, CXCR4-modified CAR-T cells have been shown to suppress MDSC recruitment by disrupting the STAT3/NF-κB/SDF-1α axis, further highlighting the therapeutic potential of targeting this axis to enhance immunotherapy [45] (see Figure 1).

### 2.3. The DDR2–STAT3–CCL20 Positive Feedback Loop in Chemoresistant HCC

Systemic chemotherapy, particularly oxaliplatin, remains a cornerstone for advanced HCC, yet its long-term efficacy is often undermined by the paradoxical remodeling of the tumor microenvironment (TME) into a highly immunosuppressive niche [46]. A critical mechanism driving this resistance involves the Discoidin Domain Receptor 2 (DDR2). In oxaliplatin-resistant HCC, DDR2 autophosphorylation triggers STAT3 Tyr705 phosphorylation, establishing a self-amplifying DDR2–STAT3 positive feedback loop that sustains tumor survival and immune evasion. Once activated, STAT3 directly binds to the promoters of CD274 (encoding PD-L1) and the chemokine CCL20, significantly upregulating their expression. While PD-L1 provides a direct inhibitory signal to T cells, CCL20 acts as a potent chemoattractant that selectively recruits CCR6^+^ PMN-MDSCs into the TME, further quenching antitumor immunity. Clinical data confirm that activation of this loop correlates with dense MDSC infiltration and poorer prognosis in HCC patients [47]. Pharmacological disruption of the DDR2–STAT3 axis abrogates PMN-MDSC chemotaxis and restores the activity of granzyme B^+^ CD8^+^ T cells. Furthermore, neutralizing CCL20 synergizes with oxaliplatin or anti-PD-L1 therapy, offering a promising “triple-hit” strategy to overcome chemoimmunotherapy resistance [13,47] (see Figure 1).

### 2.4. The β-Catenin–PF4–CXCR3 Axis and Platelet–Vascular Crosstalk

Activating mutations in CTNNB1 (encoding β-catenin), occurring in 30–40% of HCC cases, define a distinct “cold tumor” subclass characterized by an immune-desert phenotype and resistance to immune checkpoint blockade (ICB) [48,49]. Recent evidence demonstrates that hyperactivated Wnt/β-catenin signaling drives the transcriptional upregulation of platelet factor 4 (PF4/CXCL4), which serves as a selective chemoattractant for PMN-MDSCs via the CXCR3 receptor [48]. Interestingly, the impact of PF4 is dictated by the differential expression of CXCR3 isoforms: its affinity for CXCR3-A on myeloid cells facilitates MDSC recruitment and immunosuppression, while its interaction with CXCR3-B on the endothelium triggers anti-angiogenic signaling, creating a unique microenvironment of “immunosuppressive devascularization” [48,50]. This process is further exacerbated by the platelet-rich portal circulation, which amplifies local ROS production and enhances MDSC suppressive potency [51]. Clinically, HCCs harboring CTNNB1 mutations exhibit significantly higher MDSC density and minimal CD8^+^ T-cell infiltration [48,49]. Hyperactivation of β-catenin signaling in hepatocellular carcinoma recruits myeloid-derived suppressor cells (MDSCs) via the PF4-CXCR3 axis, contributing to an immune ‘cold’ or excluded phenotype [48,52]. Therapeutic strategies targeting this pathway, such as β-catenin/CBP inhibitors (e.g., ICG-001) or agents disrupting PF4-mediated trafficking (e.g., PF4-neutralizing antibodies), have shown potential in preclinical models to reduce MDSC infiltration and may sensitize these tumors to anti-PD-1 therapy [48].

### 2.5. Emerging Mechanisms and the Roadmap to Integrated Immunotherapy

In addition to established pathways such as the DDR2–STAT3–CCL20 loop (detailed in Section 2.3), emerging mechanisms in MDSC biology offer further opportunities for integrated immunotherapy in HCC. For instance, recent studies highlight the role of systemic chemotherapy in paradoxically remodeling the TME into an immunosuppressive niche [46]. Pharmacological disruption of such loops, including neutralizing CCL20, has shown potential to synergize with oxaliplatin or anti-PD-L1 therapy in preclinical models, suggesting a “triple-hit” strategy to overcome chemoimmunotherapy resistance [13,47] (see Figure 1). These findings underscore the need for multi-modal approaches that address MDSC redundancies.

### 2.6. Metabolic Reprogramming: A Core Pillar of MDSC Immunosuppressive Function in HCC

Metabolic reprogramming constitutes one of the most powerful mechanisms by which MDSCs sustain their expansion and enforce profound immunosuppression within the HCC tumor microenvironment. By hijacking glucose, lipid, amino acid, and iron metabolism pathways, MDSCs fuel their own proliferation while creating a metabolically hostile niche that impairs antitumor effector cells. These interconnected programs form a barrier to effective immunotherapy but offer exploitable vulnerabilities through targeted modulators, aligning with broader trends in liver cancer research where metabolic targeting of MDSCs enhances therapeutic outcomes.

#### 2.6.1. Enhanced Glycolysis and Lactate-Driven Immunosuppression

Hyperactivated glycolysis represents a key metabolic hallmark of tumor-associated myeloid-derived suppressor cells (MDSCs) and serves as a major mechanism sustaining their immunosuppressive functions in hepatocellular carcinoma (HCC). In the hypoxic and cytokine-rich tumor microenvironment (TME), tumor-derived factors (including hyaluronan fragments and inflammatory mediators) activate signaling pathways such as NF-κB and JAK–STAT3, which upregulate critical glycolytic enzymes, particularly hexokinase-2 (HK2) and 6-phosphofructo-2-kinase/fructose-2,6-bisphosphatase 3 (PFKFB3). This enforces aerobic glycolysis (Warburg effect), leading to abundant lactate production and its extrusion into the extracellular space [53,54].

The resulting TME acidification impairs CD8^+^ T-cell metabolism, proliferation, and cytotoxic function, while pro-tecting MDSCs from oxidative stress through pentose phosphate pathway-derived NADPH and phosphoe-nolpy-ruvate [54]. In preclinical orthotopic HCC models and patient-derived systems, pharmacological inhibition of PFKFB3 (e.g., using 3PO or derivatives like PFK-015/PFK-158) or upstream regulators markedly reduces MDSC suppressive activity, lowers intratumoral lactate levels, and restores CD8^+^ T-cell effector functions [55,56]. When combined with PD-1/PD-L1 blockade, these interventions yield synergistic tumor regression, effectively converting immunologically “cold” tumors into responsive ones, with superior outcomes compared to monotherapy [55].

These findings position glycolysis not only as an energy pathway but as a central immunosuppressive switch in MDSCs. Given the preferential overexpression of PFKFB3 in tumor-infiltrating myeloid cells relative to resting T cells, it offers a selective therapeutic target. PFKFB3 inhibitors (such as PFK-158, which completed phase I trials in advanced solid tumors showing safety and preliminary anticancer activity) and related agents continue to be investigated for their potential to reprogram the HCC metabolic landscape and sensitize primary immune check-point blockade non-responders to durable responses [57].

#### 2.6.2. Lipid Metabolism and the FATP2–PGE_2_ Axis

Aberrant lipid metabolism, particularly in polymorphonuclear MDSCs (PMN-MDSCs), constitutes a second, non-redundant pillar of immunosuppressive reprogramming in HCC. Tumor-derived GM-CSF and other cytokines induce striking overexpression of fatty acid transport protein 2 (FATP2) on the MDSC surface. FATP2 selectively imports exogenous arachidonic acid, which is rapidly converted by upregulated cyclooxygenase-2 (COX-2) into prostaglandin E_2_ (PGE_2_)—one of the most potent soluble mediators of T-cell and NK-cell paralysis [58]. Secreted PGE_2_ not only directly inhibits effector lymphocyte function locally but also propagates systemic immunosuppression via the bloodstream. Concurrently, MDSCs dramatically increase mitochondrial fatty acid oxidation (FAO) to meet the enormous energetic and reductive demands of sustained proliferation, survival, and effector molecule synthesis.

**Figure 2:**
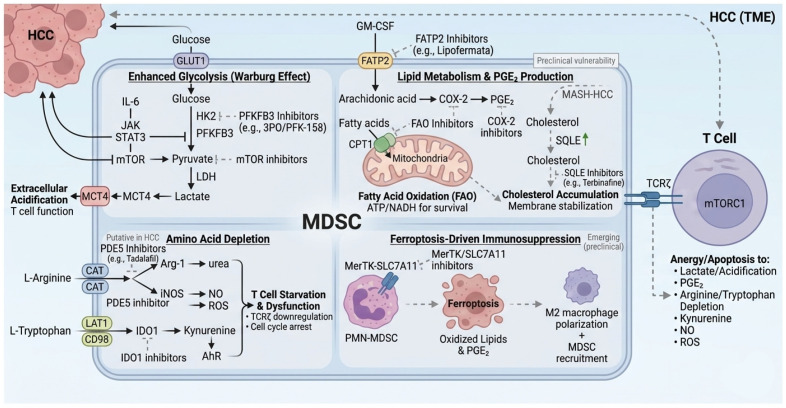
Metabolic Reprogramming of MDSCs in HCC. Overview of the key metabolic pathways reprogrammed in MDSCs within the HCC tumor microenvironment, including enhanced glycolysis (Warburg effect), lipid metabolism and PGE_2_ production (FATP2–FAO axis), amino acid depletion (Arg-1/IDO1), ferroptosis-driven immunosuppression, and cholesterol biosynthesis (SQLE). Green arrows indicate therapeutic activation/promotion. Gray dashed lines denote emerging, putative, or preclinical links (e.g., ferroptosis circuit, SQLE in MASH-HCC, spleen–liver axis), while solid lines represent more established hallmarks (see Section 2.6).

This heightened FAO is supported by upregulated carnitine palmitoyltransferase 1 (CPT1) and generates the ATP and NADH required for continuous Arg-1 and ROS production. Across multiple tumor models, pharmacological inhibition of FAO with etomoxir or genetic/pharmacological blockade of FATP2 with lipofermata rapidly abolishes MDSC suppressive activity, restores T-cell cytokine production and cytotoxicity, and markedly sensitizes tumors to PD-1/PD-L1 blockade [58,59]. In the lipid-rich, hypoxic HCC microenvironment—often exacerbated by underlying metabolic dysfunction-associated steatohepatitis—the FATP2–PGE_2_–FAO axis operates at maximal intensity, creating an almost impenetrable barrier to effective immunotherapy. However, its very specificity offers an exquisite therapeutic window: both FATP2 and FAO inhibitors exhibit minimal toxicity to resting T cells or other immune compartments. Their integration into current first-line regimens (e.g., atezolizumab–bevacizumab) or next-generation triplets represents one of the most immediately actionable strategies to dismantle lipid-driven MDSC immunosuppression and substantially broaden the fraction of HCC patients achieving deep and durable responses (see Figure 2).

#### 2.6.3. Amino Acid Depletion: Arg-1- and IDO1-Mediated Nutrient Starvation

Amino acid catabolism represents a third, non-redundant arm by which MDSCs enforce a state of nutritional deprivation that is lethal to antitumor effector cells. Monocytic MDSCs (M-MDSCs) characteristically overexpress arginase-1 (Arg-1) and inducible nitric oxide synthase (iNOS), which together rapidly deplete extracellular L-arginine. Many MDSC subsets also upregulate indoleamine 2,3-dioxygenase 1 (IDO1), irreversibly catabolizing L-tryptophan into kynurenine and downstream aryl hydrocarbon receptor (AhR) ligands [59]. The combined loss of these two essential amino acids triggers profound T-cell dysfunction: L-arginine starvation prevents mTORC1 activation, arrests the cell cycle in G_0_–G_1_, and downregulates the TCR ζ-chain, whereas tryptophan depletion and kynurenine–AhR signaling induce T-cell anergy, apoptosis, and regulatory T-cell differentiation (see Figure 2).

Clinically, this “double starvation” circuit is one of the strongest correlates of primary ICB resistance in HCC. Repurposed agents have already demonstrated promising efficacy. The PDE5 inhibitor tadalafil, by elevating cGMP, directly suppresses both Arg-1 and iNOS expression while reducing ROS production in human and murine MDSCs. In orthotopic HCC models, The PDE5 inhibitor tadalafil demonstrated >50% complete regression in orthotopic HCC models when combined with anti-PD-1 [60]. Phase I/II data in other cancers support MDSC reduction, but dedicated HCC trials are needed [61]. Preliminary preclinical data in HCC models and clinical evidence from other cancers suggest that tadalafil or similar PDE5 inhibitors may warrant exploration in combination trials for HCC, though dedicated Phase II/III studies are needed to assess safety and efficacy in this population. (see Figure 2).

#### 2.6.4. Ferroptosis-Driven Immunosuppressive Circuits in PMN-MDSCs

Emerging evidence reveals that programmed cell death pathways within MDSCs themselves can paradoxically amplify immunosuppression [62,63]. In the HCC TME, PMN-MDSCs are highly susceptible to ferroptosis—an iron-dependent, lipid-peroxidation-driven form of death. Upon undergoing ferroptosis, these cells release large quantities of oxidized phospholipids and PGE_2_, both of which act as potent “find-me” and “eat-me” signals that recruit additional MDSCs and polarize macrophages toward an M2 phenotype [62,64]. This ferroptotic cascade establishes a self-perpetuating immunosuppressive loop: dying PMN-MDSCs seed the microenvironment with oxidized lipids that further inhibit TCR signaling in residual T cells and sustain MDSC accumulation. The MerTK–SLC7A11 axis emerges as the critical rheostat protecting MDSCs from ferroptosis and maintaining ICB resistance. Genetic or pharmacological disruption of either MerTK or SLC7A11 triggers massive ferroptotic death of PMN-MDSCs, collapses the oxidized-lipid/PGE_2_ feedback circuit, and may restore responsiveness to anti-PD-L1/PD-1 therapy in otherwise refractory HCC models [65]. Thus, rather than simply eliminating MDSCs, controlled induction of ferroptosis selectively within the PMN-MDSC compartment offers a promising “kill-and-reprogram” strategy that simultaneously depletes these suppressors and harnesses their death to generate immunostimulatory signals (see Figure 2).

#### 2.6.5. Cholesterol Biosynthesis and the SQLE Node

In HCC arising on the background of metabolic dysfunction-associated steatohepatitis (MASH), chronic dyslipidemia drives profound reprogramming of de novo cholesterol biosynthesis within both tumor cells and infiltrating myeloid populations. Squalene epoxidase (SQLE), the rate-limiting enzyme downstream of HMG-CoA reductase, is markedly upregulated and emerges as a critical metabolic vulnerability that sustains the immunosuppressive phenotype of MDSCs [66]. A landmark 2024 study in Gut demonstrated that SQLE hyperactivity is a hallmark of MASH-derived HCC and directly supports MDSC accumulation through increased membrane cholesterol content, enhanced lipid raft formation, and stabilization of suppressive surface receptors. Genetic SQLE knockout or pharmacological inhibition with terbinafine was associated with reduced intratumoral MDSC accumulation and enhanced responsiveness to anti-PD-1 therapy in preclinical MASH-derived HCC models that were previously refractory to anti-PD-1 [66]. Notably, terbinafine exhibited negligible toxicity to effector T cells, highlighting an exceptionally wide therapeutic index. These findings suggest a potential link between host metabolic dysregulation and tumor immune evasion that warrants further mechanistic and clinical validation, a precision-medicine strategy for the rapidly growing subset of metabolism-driven HCCs (see Figure 2).

#### 2.6.6. Emerging 2025 Perspectives: The Spleen–Liver Axis and Integrated TME Strategies

Cutting-edge 2025 studies have illuminated the spleen–liver axis as a previously underappreciated systemic regulator of MDSC biology in obesity-associated liver disease and HCC. In high-fat diet and MASH models, splenic extramedullary hematopoiesis generates massive pools of immunosuppressive myeloid precursors that continuously seed the liver via the portal circulation. Strong positive correlations between splenic and hepatic MDSC frequencies suggest that targeting splenic myelopoiesis may represent a powerful upstream strategy to deplete tumor-promoting MDSCs at their source [50] (see Figure 2). Comprehensive TME atlases further emphasize that metabolic reprogramming rarely operates in isolation: hypoxia, lipid overload, and amino acid deprivation converge on shared downstream nodes (HIF-1α, SREBP, mTORC1) that coordinately sustain MDSC identity. Multimodal regimens that simultaneously inhibit glycolysis, FAO, cholesterol synthesis, and ferroptosis resistance—while normalizing vasculature and delivering checkpoint blockade—are now demonstrating unprecedented synergy in preclinical models previously deemed incurable [67].

Collectively, these interconnected metabolic programs form a near-impenetrable barrier to effective immunotherapy. However, their very specificity renders them exquisitely vulnerable to targeted intervention. Repurposed (tadalafil) and novel (SQLE, FATP2) metabolic modulators—often with decades of established safety data—offer immediate, low-toxicity opportunities to dismantle this barrier and Potentially broaden the therapeutic window of ICB and adoptive cell therapies in HCC (see Figure 2).

## 3. Emerging Therapeutic Strategies Targeting MDSCs in HCC

Emerging therapeutic strategies to overcome MDSC-mediated immunosuppression in hepatocellular carcinoma (HCC) encompass a multifaceted approach, including signaling pathway inhibition, metabolic modulation, epigenetic regulation, anti-angiogenic-immunotherapy combinations, transcriptional reprogramming, hematopoietic suppression, and locoregional-systemic triplet therapies. These interventions aim to disrupt MDSC recruitment, survival, and function while enhancing antitumor immunity, often in synergy with immune checkpoint blockade (ICB). By addressing the interconnected mechanisms of MDSC biology, these strategies offer promising avenues to convert immunologically “cold” tumors into responsive ones, broadening the efficacy of immunotherapy in HCC. An overview of the pharmacological agents discussed in this section, categorized by their mechanism of action and clinical development status, is provided in Table 2.

The following table provides an overview of all pharmacological agents discussed in this review, categorized by their primary nature/type and role in modulating MDSC biology or function within the HCC tumor microenvironment. Mechanisms are focused on MDSC-related effects as described in Section 3, with many agents showing promise in preclinical models and/or early-phase clinical trials (detailed status for select agents is provided in subsequent tables).

Overview of selected pharmacological agents discussed in this review, categorized by nature/type and their proposed role/mechanism in modulating MDSC biology or function within the HCC tumor microenvironment, with supporting references.

### 3.1. Signaling Pathway Inhibition: STAT3 as the Central Target

**Figure 3:**
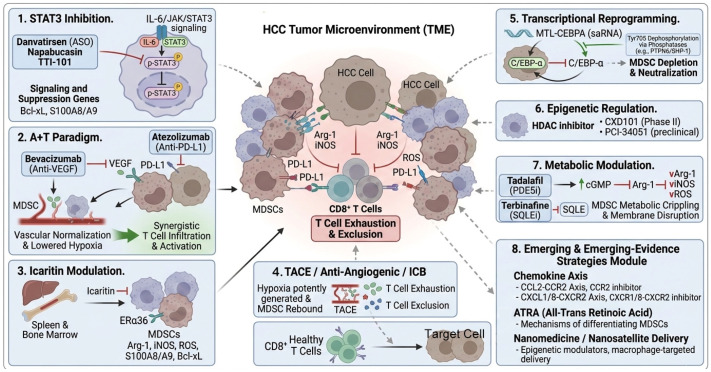
MDSC-Targeted Therapeutic Strategies in Hepatocellular Carcinoma (HCC). Integrated schematic of established and emerging therapeutic approaches targeting MDSC-mediated immunosuppression in HCC, including STAT3 inhibition, A + T paradigm, icaritin modulation, locoregional-systemic triplet therapy, transcriptional reprogramming, epigenetic regulation, metabolic modulation, chemokine axis blockade, ATRA-induced differentiation, and nanomedicine strategies. Solid arrows represent established or stimulatory pathways, while green arrows indicate therapeutic activation/promotion. Gray dashed lines indicate emerging, preclinical, early-phase, or conceptually supported links requiring further validation. (see Section 3.1, Section 3.2, Section 3.3, Section 3.4, Section 3.5, Section 3.6, Section 3.7, Section 3.8, Section 3.9, Section 3.10 and Section 3.11).

Persistent activation of signal transducer and activator of transcription 3 (STAT3) represents a central regulatory pathway governing MDSC expansion, blockade of myeloid maturation, and acquisition of immunosuppressive functions in hepatocellular carcinoma (HCC) [68]. Within the HCC TME, STAT3 directly transactivates arginase-1 (Arg-1) and other suppressive effectors while sustaining anti-apoptotic programs (e.g., Bcl-xL) and recruiting additional immunosuppressive populations through S100A9 and related mediators [69,70]. Consequently, pharmacological STAT3 inhibition can simultaneously induce MDSC apoptosis, promote their differentiation into non-suppressive mature myeloid cells (macrophages and dendritic cells), and relieve T-cell exhaustion, thereby restoring antitumor immunity. (see Figure 3).

Mechanistically, STAT3 blockade primarily disrupts the upstream IL-6/JAK signaling axis, preventing phosphorylation and nuclear translocation of STAT3. This leads to downregulation of multiple downstream targets critical for MDSC biology, including anti-apoptotic proteins, proliferation drivers, and chemokines responsible for MDSC recruitment. Several STAT3-directed agents have progressed to clinical evaluation in HCC: Danvatirsen (AZD9150), a next-generation antisense oligonucleotide, efficiently depletes STAT3 mRNA. Early phase trials in heavily pretreated lymphomas and solid tumors demonstrated antitumor activity and acceptable tolerability [71]. Its combination with the anti-PD-L1 antibody durvalumab is currently under investigation in HCC expansion cohorts, building on preclinical evidence of synergistic MDSC depletion [31] (see Figure 3).

Napabucasin (BBI-608), despite failing phase III testing in colorectal cancer due to insufficient survival benefit, retains preclinical promise in HCC through dual inhibition of STAT3 and cancer stemness pathways [37]. Nano-formulation strategies are being explored to improve bioavailability and reduce off-target effects. TTI-101, a novel, highly selective oral STAT3 inhibitor, has shown particular promise. In a phase I trial (NCT03195699) involving 17 heavily pretreated advanced HCC patients, TTI-101 monotherapy achieved an 18% objective response rate with a favorable safety profile. The ongoing Phase 1b/2 REVERT-Liver Cancer trial (NCT05440708) assesses TTI-101 monotherapy or in combination with pembrolizumab or atezolizumab plus bevacizumab in advanced HCC, targeting STAT3-driven immunosuppression [72].

STAT3 inhibition offers pleiotropic benefits in HCC: it directly impairs tumor-cell proliferation, invasion, and stemness while reprogramming the immunosuppressive myeloid compartment. However, on-target bone-marrow toxicity remains a concern with prolonged exposure. Next-generation approaches, including nanoparticle-mediated delivery and rational combination regimens, are expected to enhance therapeutic index while minimizing hematologic adverse events [67]. In summary, STAT3 represents one of the most validated and clinically actionable nodes for overcoming MDSC-driven resistance in HCC, with multiple agents now in advanced-stage testing. Complementing STAT3, colony-stimulating factor 1 receptor (CSF1R) signaling sustains MDSC and TAM survival in the TME [73]. CSF1R inhibitors (e.g., pexidartinib) reduce MDSC infiltration in preclinical HCC models by depleting CSF1-dependent myeloid populations [74,75]. A phase 1 trial (NCT02718911) combining a CSF1R inhibitor (LY3022855) with anti-PD-L1 (durvalumab) in advanced solid tumors showed acceptable safety but limited clinical activity, though liver toxicity requires monitoring with this class of agents [76]. CSF1R blockade synergizes with ICB by alleviating MDSC-mediated suppression, offering a broader myeloid-targeting approach [77].

### 3.2. Metabolic Modulation: Exploiting MDSC-Specific Metabolic Vulnerabilities

In addition to direct interference with signaling pathways, targeting the distinctive metabolic dependencies of MDSCs has emerged as one of the most promising and rapidly translatable therapeutic avenues in HCC. These cells rely on highly reprogrammed metabolic circuits to sustain both their own survival and their potent immunosuppressive activity. By selectively disrupting these circuits, it is possible to simultaneously impair MDSC function and restore antitumor immunity with agents that already possess favorable safety profiles (see Figure 3). A prime example is tadalafil, a phosphodiesterase-5 (PDE5) inhibitor long used clinically for other indications. Tadalafil elevates intracellular cGMP, which in turn suppresses the expression of arginase-1 and inducible nitric oxide synthase while reducing reactive oxygen species production—the core mechanisms underlying MDSC-mediated T-cell inhibition [78]. A landmark 2023 preclinical study in orthotopic HCC models showed that tadalafil not only directly neutralizes MDSC suppressive activity but also abrogates hypoxia-driven STAT3 activation, leading to a striking decrease in intratumoral MDSC accumulation. When combined with anti-PD-1 blockade, this resulted in 55% tumor growth inhibition and robust CD8^+^ T-cell infiltration and activation [66]. Supporting evidence from patients with head-and-neck squamous cell carcinoma demonstrated that tadalafil significantly lowers circulating MDSC and regulatory T-cell numbers while enhancing tumor-specific immune responses [67]. Given its oral bioavailability, established long-term safety profile, and dual impact on both MDSC metabolism and STAT3 signaling, tadalafil represents one of the most promising and readily translatable partners for immune checkpoint therapy in MDSC-rich HCC [67] (see Figure 3).

Another compelling target lies in cholesterol biosynthesis, particularly in the rapidly growing subset of HCCs arising from metabolic dysfunction-associated steatohepatitis (MASH). In these tumors, hyperactivation of de novo cholesterol synthesis sustains the immunosuppressive phenotype of MDSCs [64]. Squalene epoxidase (SQLE), the rate-limiting enzyme of this pathway, is markedly overexpressed. A 2024 study published in Gut provided compelling proof-of-concept evidence: genetic ablation or pharmacological inhibition of SQLE using terbinafine significantly reshaped intratumoral lipid metabolism, reduced MDSC infiltration and immunosuppressive activity, and restored sensitivity to anti-PD-1 therapy in previously refractory MASH-derived HCC models [61,66]. This work elegantly links host dyslipidemia to tumor immune evasion and offers a precision approach for metabolism-driven HCC. Collectively, metabolic modulators such as tadalafil and SQLE inhibitors exploit hard-wired vulnerabilities unique to MDSCs while minimizing systemic toxicity. Their seamless integration into existing ICB regimens—either as repurposed drugs or optimized next-generation analogs—represents one of the most clinically feasible strategies to dismantle MDSC-driven resistance and broaden the therapeutic window of immunotherapy in hepatocellular carcinoma.

### 3.3. Epigenetic Regulation: HDAC Inhibitors as Potent MDSC-Reprogramming Agents

Epigenetic modifiers, particularly histone deacetylase (HDAC) inhibitors, have emerged as powerful tools to reverse MDSC-mediated immunosuppression by simultaneously targeting multiple layers of the tumor immune microenvironment. Rather than directly killing MDSCs, these agents reprogram their recruitment, survival, and function while sensitizing tumor cells to immune attack, making them ideal partners for immune checkpoint blockade. HDAC inhibitors suppress the accumulation and activity of monocytic MDSCs and enhance the cytotoxicity of natural killer cells through upregulation of activating receptors and effector pathways [79,80] (see Figure 3).

More selective agents have revealed even broader potential in HCC. The novel selective class-I HDAC inhibitor CXD101 triggers tumor-cell pyroptosis via activation of the STAT1–gasdermin E (GSDME) axis, transforming immunologically “cold,” HDAC-overexpressing HCCs into inflamed, ICB-responsive lesions and generating long-lived CD8^+^ T-cell memory [81]. It also reduces intratumoral MDSC infiltration and shifts the myeloid compartment toward an immunostimulatory phenotype. Building on these findings, an ongoing phase II trial (NCT05873244) is evaluating CXD101 combined with PD-1 blockade in patients with advanced HCC who progressed on or were refractory to first-line atezolizumab–bevacizumab [81].

Selective HDAC8 inhibitors (e.g., PCI-34051) reprogram the tumor microenvironment (TME) by increasing histone H3K27 acetylation in HCC cells, thereby reactivating T cell–trafficking chemokines (e.g., CCL4), enhancing CD8^+^ T cell infiltration, and reducing regulatory T cells. In preclinical HCC models, HDAC8 blockade markedly potentiates anti-PD-1/PD-L1 efficacy, leading to increased tumor control and durable responses in otherwise refractory tumors [82]. By simultaneously disrupting epigenetic programs that sustain MDSC identity, inducing inflammatory tumor-cell death, and relieving T-cell exclusion, HDAC inhibitors achieve multifaceted remodeling of the immunosuppressive milieu. Their favorable toxicity profile and synergy with existing standards of care position them as one of the most versatile and clinically advanced classes of MDSC-targeting agents in hepatocellular carcinoma.

### 3.4. Anti-Angiogenic–Immunotherapy Synergy: Bevacizumab Plus Atezolizumab as a Paradigm

The combination of bevacizumab (anti-VEGF) and atezolizumab (anti-PD-L1), widely known as “A + T”, represents one of the most clinically established and successful strategies currently available for addressing MDSC-driven resistance in advanced HCC. Vascular endothelial growth factor (VEGF) is not merely an angiogenic driver; it functions as a potent immunosuppressive cytokine that directly expands MDSCs, promotes their Arg-1 and PD-L1 expression, and polarizes tumor-associated macrophages toward an M2 phenotype through STAT3-dependent mechanisms. Chronic VEGF signaling also disrupts endothelial adhesion molecules, impairing CD8^+^ T-cell trafficking into the tumor core. Bevacizumab normalizes pathological tumor vasculature, reduces hypoxia, and rapidly decreases circulating and intratumoral MDSC frequencies while lowering Treg infiltration. This vascular reprogramming creates a permissive microenvironment that dramatically enhances the efficacy of atezolizumab [83]. The pivotal IMbrave150 trial established A + T as the global first-line standard, demonstrating a median overall survival of 19.2 months versus 13.4 months with sorafenib, alongside superior progression-free survival (6.9 vs. 4.3 months) and objective response rates [8]. Long-term follow-up and multiple real-world cohorts have consistently confirmed durable benefit, manageable safety, and quality-of-life advantages, even in patients with high MDSC burden at baseline [84]. By simultaneously blocking a major upstream driver of MDSC expansion and relieving physical barriers to T-cell infiltration, the A + T regimen achieves profound and sustained reprogramming of the HCC immune landscape. It remains the benchmark against which all emerging MDSC-targeted combinations are measured and continues to serve as the backbone for next-generation triplet and quadruplet regimens (see Figure 3).

### 3.5. Transcriptional Reprogramming with MTL-CEBPA

MTL-CEBPA is an innovative small activating RNA (saRNA) that restores expression of the liver-enriched transcription factor C/EBP-α, which is frequently downregulated during hepatocarcinogenesis and chronic liver disease. Loss of C/EBP-α not only promotes tumor-cell de-differentiation and stemness but also sustains persistent STAT3 phosphorylation in the myeloid compartment, thereby driving massive MDSC expansion and reinforcing their immunosuppressive phenotype [85,86]. Mechanistically, restored C/EBP-α directly interacts with STAT3 by competing for binding sites or recruiting phosphatases (e.g., PTPN6/SHP-1), leading to STAT3 dephosphorylation at Tyr705 and inhibition of its nuclear translocation. This antagonizes pro-MDSC STAT3 signaling, downregulating downstream targets like Bcl-xL (anti-apoptotic) and S100A9 (recruitment), resulting in MDSC apoptosis, reduced expansion, and functional neutralization (e.g., lower Arg-1/ROS) [86]. By selectively upregulating C/EBP-α in both hepatocytes and myeloid cells, MTL-CEBPA exerts a powerful dual effect: it redifferentiates malignant hepatocytes, suppresses cancer stem-cell properties, and—most importantly for immunotherapy—interrupts STAT3 signaling, leading to rapid depletion of intratumoral MDSCs, reduced production of Arg-1 and ROS, and a marked shift toward immunostimulatory myeloid subsets [85,86] (see Figure 3).

Preclinical orthotopic HCC models have repeatedly demonstrated that C/EBP-α restoration dramatically decreases MDSC infiltration, enhances CD8^+^ T-cell trafficking and effector function, and sensitizes otherwise resistant tumors to PD-1/PD-L1 blockade. This synergy arises from C/EBP-α’s disruption of the STAT3-MDSC feedback loop, converting “cold” tumors into inflamed lesions responsive to ICB. The first-in-human phase I OUTREACH trial, conducted primarily in advanced HCC (94% of patients), confirmed excellent tolerability of intravenous MTL-CEBPA and provided striking early efficacy signals: a disease control rate of 41% was achieved, including one patient with sorafenib-refractory disease who experienced a confirmed partial response lasting 22.3 months [85]. This durable response in a heavily pretreated individual underscores the potential of transcriptional reprogramming to overcome both tumor-intrinsic and MDSC-driven resistance mechanisms. With its favorable safety profile, liver-specific activity, and ability to simultaneously target oncogenic pathways and the immunosuppressive myeloid compartment, MTL-CEBPA represents one of the most elegant MDSC-focused strategies currently in clinical development. Ongoing and planned combination studies with immune checkpoint inhibitors, multikinase inhibitors, and locoregional therapies aim to establish it as a cornerstone of next-generation HCC immunotherapy regimens capable of converting immunologically cold tumors into durable responders (see Figure 3).

### 3.6. Hematopoietic Suppression and Direct MDSC Modulation: Icaritin

Icaritin (icaritin soft capsules), approved by China’s National Medical Products Administration (NMPA) in January 2022 as the first small-molecule immunomodulator specifically for advanced hepatocellular carcinoma in China, represents a landmark achievement in clinically validated MDSC-targeted therapy. This natural flavonoid derivative selectively binds membrane-bound estrogen receptor ERα36 on myeloid precursors and tumor-associated macrophages, thereby interrupting the IL-6/JAK2/STAT3 signaling cascade at its earliest steps [87]. Specifically, ERα36 inactivation prevents IL-6-mediated JAK2 phosphorylation, blocking downstream STAT3 Tyr705 phosphorylation and nuclear translocation. This disrupts pro-MDSC STAT3 signaling, leading to downregulation of immunosuppressive effectors (e.g., Arg-1, iNOS, ROS) and inhibition of genes promoting MDSC survival and recruitment (e.g., Bcl-xL, S100A8/A9) [87,88]. The downstream consequences are profound: rapid dephosphorylation of STAT3, marked downregulation of Arg-1, iNOS, and reactive oxygen species production within MDSCs, and functional neutralization of their immunosuppressive activity (see Figure 3). Unlike conventional cytotoxic or broad immunosuppressive agents, icaritin exerts a highly selective “source-and-function” double hit. At the systemic level, it potently inhibits extramedullary hematopoiesis in the spleen and liver, dramatically reducing the generation of CD11b^+^ Gr-1^+^ myeloid precursors that would otherwise differentiate into tumor-promoting MDSCs [88]. Simultaneously, residual MDSCs are redirected toward mature, non-suppressive macrophage and dendritic-cell lineages while losing their ability to impair CD8^+^ cytotoxic T lymphocytes (CTLs). In orthotopic and patient-derived xenograft HCC models, icaritin monotherapy induces striking reductions in splenic and intratumoral MDSC density, restores IFN-γ production by tumor-infiltrating CTLs, and significantly prolongs survival [89] (see Figure 3).

### 3.7. Locoregional–Systemic Triplet Therapy: TACE Combined with Anti-Angiogenic Agents and Immune Checkpoint Blockade

Transarterial chemoembolization (TACE) remains the cornerstone of locoregional therapy for intermediate-stage hepatocellular carcinoma (HCC), yet its long-term efficacy is limited by rapid reconstitution of an immunosuppressive microenvironment, with myeloid-derived suppressor cells (MDSCs) playing a central role in driving immune evasion and tumor progression in HCC [90]. TACE initially triggers massive tumor necrosis and release of neoantigens, which transiently reduces monocytic MDSC frequencies and activates effector T cells. However, the ensuing hypoxia potently upregulates HIF-1α and VEGF, driving explosive re-recruitment of MDSCs—a key mechanism underlying HCC-specific immune tolerance and resistance to subsequent therapies [90,91,92]. This MDSC rebound not only quenches antitumor immunity but also reinforces the tolerogenic liver environment characteristic of HCC, leading to rapid disease progression and diminished response to immune checkpoint blockade (ICB) (see Figure 3).

Triple combinations that couple TACE with VEGF blockade and PD-1/PD-L1 inhibition were designed to exploit the antigen-releasing benefit of TACE while simultaneously preventing MDSC resurgence, thereby dismantling the MDSC-dominated immunosuppressive barrier in HCC. Anti-VEGF agents (bevacizumab or multitargeted TKIs such as lenvatinib) normalize tumor vasculature, alleviate hypoxia, and directly suppress VEGF-driven MDSC expansion and M2 polarization [83]. Concurrent PD-1/PD-L1 blockade then capitalizes on the improved T-cell trafficking and restored antigen presentation, ultimately overcoming MDSC-mediated T-cell exhaustion—a hallmark of HCC immunotherapy resistance. Two landmark phase III trials have now provided validation for this triplet strategy, though with mixed results. The EMERALD-1 trial showed that the addition of durvalumab (anti-PD-L1) and bevacizumab to TACE significantly extended progression-free survival compared with TACE alone (15.0 versus 8.2 months; HR 0.77, *p* = 0.032), successfully meeting the primary endpoint and demonstrating a strong trend toward overall survival improvement [93]. Similarly, the LEAP-012 trial, which combined TACE with lenvatinib and pembrolizumab (anti-PD-1), achieved a clinically meaningful prolongation of progression-free survival (14.6 versus 10.0 months; HR 0.66, *p* = 0.0002) but did not meet the prespecified overall survival endpoint at interim analysis (HR 0.80, *p* = 0.087), leading to study closure in October 2025 due to low likelihood of achieving statistical significance in future analyses. No new safety signals were observed [94]. Recent trials like EMERALD-1 show promise for TACE combined with anti-angiogenic agents and ICB in improving outcomes (e.g., PFS benefits), though LEAP-012 did not meet OS endpoints at interim analysis and was closed [94]. This approach represents a promising but evolving strategy, with further Phase III data needed to establish it as a standard of care. Particularly for patients with unresectable, non-metastatic hepatocellular carcinoma at high risk of post-TACE MDSC-driven immune escape [95]. By transforming a historically locoregional procedure into a potential immunological priming event—through antigen release and modulation of the TME—and combining it with agents that may suppress MDSC-mediated immunosuppression, these triplet regimens (TACE + anti-angiogenic + ICB) have demonstrated clinically meaningful improvements in progression-free survival in recent phase III trials (e.g., EMERALD-1 and LEAP-012). While overall survival data remain immature and have not consistently shown statistical superiority over TACE alone, these approaches hold promise for enhancing disease control in selected patients with intermediate-stage HCC. This evolving strategy could serve as a platform for future multimodal regimens incorporating additional agents targeting MDSC recruitment or function, pending further validation in ongoing and planned studies.

### 3.8. Targeting Chemokine Axes: CCR2/CCL2 and CXCR2 Inhibitors

Chemokine axes drive MDSC recruitment to the HCC TME, making them key targets for disrupting infiltration as part of emerging therapeutic strategies targeting MDSCs in HCC. These pathways not only facilitate the homing of immunosuppressive myeloid cells but also amplify tumor progression by sustaining an immunosuppressive niche that limits T-cell effector functions. The CCL2-CCR2 axis is central: tumor-derived CCL2 recruits CCR2^+^ M-MDSCs, enhancing Arg-1 and TGF-β-mediated suppression [96,97]. This recruitment process is particularly pronounced in HCC, where chronic inflammation and fibrosis exacerbate MDSC accumulation, leading to resistance against immune checkpoint blockade (ICB). CCR2 inhibitors reduce MDSC accumulation in preclinical HCC models, synergizing with anti-PD-1 to improve survival [23,98]. For instance, in murine HCC models, blocking CCR2 has been shown to reprogram the TME by decreasing M-MDSC infiltration and increasing CD8^+^ T-cell activation, thereby enhancing overall antitumor immunity and potentially delaying tumor recurrence post-resection.

Similarly, the CXCL1/8-CXCR2 axis recruits PMN-MDSCs, correlating with poor prognosis [99]. This axis is upregulated in HCC patients with advanced disease stages, where PMN-MDSCs contribute to vascular invasion and metastasis through elevated ROS and neutrophil extracellular traps (NETs). CXCR2 inhibitors (e.g., SX-682) block this in HCC xenografts, decreasing ROS production and sensitizing to ICB [100,101]. Preclinical studies demonstrate that CXCR2 blockade not only depletes PMN-MDSCs but also disrupts their cross-talk with tumor cells, reducing HCC proliferation and invasiveness. Ongoing trials evaluate CXCR2 blockade with pembrolizumab, demonstrating potential to overcome resistance in MDSC-high tumors [102]. These inhibitors offer low-toxicity options for combination regimens, positioning them as promising adjuncts to standard HCC therapies like sorafenib or lenvatinib, where MDSC targeting could enhance response rates in ICB-refractory patients.

### 3.9. Promoting MDSC Differentiation: All-Trans Retinoic Acid (ATRA)

ATRA induces MDSC differentiation into mature, non-suppressive dendritic cells and macrophages by activating ERK signaling and downregulating ROS/Arg-1 [103,104], representing an innovative approach in emerging therapeutic strategies targeting MDSCs in HCC. This differentiation shifts MDSCs from an immature, immunosuppressive state to functional antigen-presenting cells, thereby restoring immune surveillance in the TME. In HCC models, ATRA reduces MDSC frequency and enhances NK/T-cell activity [4,105], with additional benefits including inhibition of tumor angiogenesis and fibrosis, which are hallmarks of HCC progression driven by chronic liver disease. For example, in orthotopic HCC mouse models, ATRA treatment has led to decreased MDSC-derived suppressive factors like iNOS and IDO, fostering a more immunogenic environment that supports adaptive immune responses.

Clinical trials combining ATRA with ICB in other cancers, such as melanoma, have shown MDSC depletion and improved responses [13,106], providing a blueprint for HCC applications where similar mechanisms could address the high MDSC burden associated with poor ICB efficacy. When paired with ICB, ATRA has potential to convert “cold” tumors in HCC, warranting further studies [107]. This strategy is particularly appealing for HCC due to its vitamin A derivative nature, offering a repurposed agent with established safety profiles that could be integrated into multimodal therapies, such as combining with locoregional treatments like transarterial chemoembolization (TACE) to potentiate systemic immunotherapy.

### 3.10. Nanomedicine Strategies for Targeting the TME and MDSCs in HCC

Nanomedicine offers innovative tools to modulate the immunosuppressive tumor microenvironment (TME) in hepatocellular carcinoma (HCC), where macrophage-targeted strategies provide conceptual and technical parallels to emerging MDSC-targeted delivery approaches. Tumor-associated macrophages (TAMs), often polarized to an M2 phenotype, share key immunosuppressive functions with MDSCs, such as cytokine secretion and T-cell inhibition, and contribute to MDSC recruitment through pathways like CCL2 signaling [108]. Macrophage-targeted nanomedicines, including cell membrane-coated nanoparticles, exploit immune evasion mechanisms (e.g., CD47 expression) to selectively deliver payloads to M2 TAMs, reprogramming them toward an M1 antitumor phenotype and thereby reducing MDSC infiltration in preclinical HCC models [109,110].

Recent advances in TME-responsive nanoplatforms further demonstrate technological feasibility. For instance, hollow mesoporous manganese dioxide (HMnO_2_)-based nanoparticles respond to the acidic pH, high H_2_O_2_, and glutathione (GSH) levels characteristic of the TME, triggering controlled degradation for oxygen generation, ROS amplification, and chemodynamic therapy [111]. Although initially demonstrated in other tumor models, similar responsive designs hold potential for targeting hypoxic regions in HCC that are enriched in MDSCs and TAMs, potentially enhancing synergy with immune checkpoint blockade.

Distinctions must be maintained between proof-of-concept studies and clinically translatable MDSC-targeted delivery. Preclinical HCC nanomedicines, such as epi-immune nanosatellites co-delivering epigenetic modulators, have shown the ability to reprogram TAMs and suppress CCL2-driven MDSC recruitment in mouse models, yielding robust antitumor effects [110]. However, these remain at the conceptual stage, constrained by challenges in scalability, long-term biocompatibility, and liver-specific toxicity. Clinically viable approaches will require rigorous phase I trials focused on pharmacokinetics, safety, and liver tolerance, with ongoing TAM-targeted nanomedicines in other cancers offering a pathway forward [108,112]. Integrating nanomedicine with AI-guided patient stratification could accelerate translation and help overcome MDSC-driven resistance in HCC. Emerging nanotherapeutic strategies specifically aim to remodel the immunosuppressive microenvironment by targeting inflammation and key suppressor cells like MDSCs, with pH-responsive or multifunctional nanoparticles showing promise in reducing MDSC activity and restoring antitumor immunity in HCC models [112,113].

### 3.11. Clinical Caveats and Contraindications in Targeting MDSC-Mediated Immunosuppression in Hepatocellular Carcinoma

While emerging strategies targeting myeloid-derived suppressor cells (MDSCs) in hepatocellular carcinoma (HCC) hold significant promise, their clinical implementation requires careful consideration of safety profiles, particularly in patients with underlying cirrhosis or advanced liver disease.

In sections advocating ferroptosis induction in polymorphonuclear MDSCs (PMN-MDSCs), such as through disruption of the MerTK–SLC7A11 axis, ref. [65] potential hepatotoxicity must be noted; ferroptosis involves lipid peroxidation and oxidative stress, which could exacerbate liver injury in cirrhotic patients, leading to acute decompensation or worsening fibrosis [65,114]. Preclinical models suggest selective induction within MDSCs minimizes off-target effects, but human trials are needed to assess liver enzyme elevations and long-term outcomes.

For broad lipid metabolism blockade, including inhibition of fatty acid transport protein 2 (FATP2) or squalene epoxidase (SQLE) using agents such as lipofermata or terbinafine, potential risks include dyslipidemia, mitochondrial dysfunction, and hepatotoxicity. These arise because the targeted pathways are essential for normal hepatic lipid homeostasis, with terbinafine notably associated with rare but severe drug-induced liver injury [58,61]. Terbinafine, in particular, has been associated with idiosyncratic liver injury in up to 1 in 50,000 patients, necessitating baseline liver function monitoring and avoidance in those with Child-Pugh class C cirrhosis [115].

Microbiome modulation strategies, such as fecal microbiota transplantation (FMT) or probiotic supplementation (e.g., Akkermansia muciniphila), carry infectious risks, including pathogen transmission or sepsis in immunocompromised HCC patients undergoing immunotherapy. Antibiotic stewardship is critical, as peritherapeutic exposure can worsen dysbiosis and MDSC enrichment, potentially increasing Clostridioides difficile infection rates [116].

Additionally, STAT3 inhibitors (e.g., danvatirsen or TTI-101) may cause on-target bone marrow suppression, manifesting as neutropenia or thrombocytopenia, due to STAT3’s role in hematopoiesis [31,72]. These agents should be contraindicated in patients with baseline cytopenias or active infections.

Clinical Caveats and Contraindications: Ferroptosis induction in PMN-MDSCs risks oxidative stress-induced hepatotoxicity; contraindicate in decompensated cirrhosis (Child-Pugh B/C) [65,114]. Lipid metabolism blockers (e.g., SQLE inhibitors) may cause dyslipidemia or idiosyncratic liver injury; monitor LFTs and avoid in severe hepatic impairment [61,115]. Microbiome interventions like FMT pose infection risks; screen donors rigorously and contraindicate in immunosuppressed states or recent antibiotic use [116]. STAT3 inhibitors carry marrow toxicity; contraindicate in cytopenic patients (ANC < 1.5 × 10^9^/L or platelets < 100 × 10^9^/L) [31,72]. All strategies require multidisciplinary oversight, with phase I/II trials prioritizing safety endpoints in HCC cohorts.

## 4. Targeting the Gut–Liver Axis: Harnessing the Microbiome to Overcome MDSC-Driven Resistance

The gut–liver axis has emerged as one of the most powerful extrinsic regulators of hepatic immunity and MDSC biology in HCC. Chronic gut barrier dysfunction (“leaky gut”) permits translocation of microbial products—most notably lipopolysaccharide (LPS)—via the portal circulation. In the liver, LPS engages Toll-like receptor 4 (TLR4) on resident macrophages and myeloid precursors, triggering CXCL1–CXCR2 signaling that massively expands and activates PMN-MDSCs while reinforcing their Arg-1 and ROS-dependent suppressive activity [23,117]. Clinical studies consistently show that HCC patients with dysbiosis exhibit higher intratumoral MDSC densities, reduced tertiary lymphoid structures, and poorer responses to ICB. Conversely, a balanced microbiome rich in beneficial genera (e.g., Akkermansia, Lachnoclostridium) correlates with enhanced antitumor immunity and prolonged progression-free and overall survival after PD-1 blockade [118]. These observations have catalyzed microbiome-directed strategies aimed at depleting pathogenic drivers of MDSC expansion while enriching protective species. Oral supplementation with Akkermansia muciniphila in preclinical MAFLD-associated HCC models restores gut barrier integrity, lowers circulating LPS, and dramatically reduces monocytic MDSC and M2-macrophage infiltration. When combined with anti-PD-1 therapy, Akkermansia achieves synergistic tumor control that far exceeds either intervention alone [119]. Fecal microbiota transplantation (FMT) from ICB responders or healthy donors is now under active clinical investigation. Early phase trials such as FAB-HCC (NCT05750030), which completed enrollment in March 2025, are testing whether FMT can convert primary non-responders to the atezolizumab–bevacizumab regimen into durable responders by reshaping the myeloid compartment; full results are awaited [120,121].

Antibiotic exposure during immunotherapy, by contrast, profoundly worsens outcomes. Large real-world cohorts and meta-analyses demonstrate that peritherapeutic antibiotics shorten both overall and progression-free survival in HCC patients receiving targeted or immune-based therapies, an effect directly attributable to dysbiosis-induced TLR4–CXCR2-driven MDSC enrichment [116,122]. Emerging evidence also highlights microbiome-derived metabolites as key immunomodulators. Short-chain fatty acids (e.g., butyrate) exhibit context-dependent effects: while anti-inflammatory in autoimmune liver disease, they can paradoxically enhance MDSC suppressive function in the tumor setting via HDAC inhibition and FAO upregulation [123]. Secondary bile acids and tryptophan catabolites (e.g., kynurenine–AhR signaling) similarly reinforce MDSC accumulation and T-cell anergy [124]. Taken together, the gut–liver axis represents a clinically accessible, non-invasive lever to systemically deplete MDSCs and sensitize HCC to immunotherapy. Ongoing and future trials combining engineered probiotics, next-generation FMT, or rationally designed microbial consortia with ICB and MDSC-depleting agents hold immense promise to dramatically expand the fraction of patients achieving deep and lasting responses.

### 4.1. Negative Impact of Antibiotics on Immunotherapy Efficacy

Broad-spectrum antibiotic use is associated with disruption of the gut–liver axis and represents one of the strongest observational risk factors for immunotherapy failure, though causality remains to be established in hepatocellular carcinoma. Multiple large-scale retrospective studies and meta-analyses have demonstrated an association between peritherapeutic antibiotic exposure and inferior outcomes, including shortened OS and PFS in HCC patients receiving immunotherapy [116,122]. However, these findings are primarily observational and may be confounded by factors such as tumor burden, infection status, or baseline disease severity, warranting prospective trials to establish causality [116,125].

By depleting commensal microbial communities and collapsing microbial diversity, antibiotics trigger a cascade that directly fuels MDSC-driven immune evasion. Multiple large-scale studies have now strongly associated peritherapeutic antibiotic exposure—particularly within the 30-day window before or after initiation of immune checkpoint blockade—to markedly inferior outcomes. In real-world cohorts and multi-center retrospective analyses involving thousands of HCC patients receiving either targeted therapy or immune-based regimens, antibiotic administration consistently shortened both overall survival and progression-free survival [116,122].

Mechanistically, antibiotic-induced dysbiosis compromises intestinal barrier integrity, dramatically increasing translocation of bacterial products (primarily LPS) into the portal circulation. This surge engages hepatic TLR4 on Kupffer cells and myeloid precursors, rapidly amplifying CXCL1–CXCR2 signaling and driving explosive expansion of highly suppressive PMN-MDSCs [23,116]. The resulting surge in intratumoral MDSC density creates an insurmountable barrier to CD8^+^ T-cell infiltration and effector function, effectively converting potentially responsive tumors into primary non-responders. The clinical implications are stark: even short courses of commonly prescribed antibiotics can abrogate the benefit of otherwise effective regimens such as atezolizumab–bevacizumab or durvalumab–tremelimumab. Consequently, current guidelines and expert consensus increasingly emphasize strict antibiotic stewardship throughout the entire immunotherapy journey in HCC, reserving their use for clear infectious indications and favoring narrow-spectrum agents when absolutely necessary. Proactive microbiome-preserving strategies—such as co-administration of select probiotics or early FMT rescue—are now being explored to mitigate this iatrogenic immunosuppression and salvage therapeutic efficacy in antibiotic-exposed patients.

### 4.2. Immunomodulatory Roles of Microbiome-Derived Metabolites

Microbial metabolites serve as critical signaling molecules that bridge the gut microbiota and hepatic immune microenvironment, exerting profound and often bidirectional effects on MDSC biology in HCC. Short-chain fatty acids (SCFAs), particularly butyrate, exemplify this complexity through the so-called “butyrate paradox.” This paradox has been well-documented in colorectal cancer models and human datasets, where butyrate promotes normal colonocyte proliferation but inhibits cancer cell growth [123,126]. In HCC, human datasets from NAFLD-HCC cohorts show elevated butyrate levels correlating with immunosuppressive responses, though some studies suggest protective effects via NK cell enhancement [127,128]. Further HCC-specific prospective data are needed to resolve these context-dependent effects, as much evidence is extrapolated from other gastrointestinal cancers. In non-malignant liver inflammation, butyrate acts as a potent anti-inflammatory agent via HDAC inhibition and enhancement of MDSC fatty acid oxidation, thereby limiting tissue damage. Within the tumor context, however, the same epigenetic and metabolic reprogramming paradoxically amplifies MDSC suppressive potency, further entrenching immune evasion [129].

Secondary bile acids, generated by microbial deconjugation and dehydrogenation of primary bile acids, constitute another pivotal class. Alterations in bile acid pools—commonly observed in cirrhosis and HCC—promote MDSC accumulation through TGR5-dependent pathways and reinforce M2 macrophage polarization, collectively sustaining an immunosuppressive milieu [124]. Tryptophan catabolites, especially kynurenine produced via the IDO1–AhR axis, represent one of the most potent metabolite-driven immunosuppressive circuits. In HCC, human datasets (e.g., TCGA LIHC) show elevated kynurenine levels correlating with poor prognosis and immune suppression [130,131]. The kynurenine-AhR pathway activates AhR on MDSCs, promoting Treg differentiation and T-cell anergy, as evidenced in HCC-specific studies [132,133]. However, some mechanisms are extrapolated from glioma or colorectal cancer models, where AhR signaling similarly reinforces immunosuppression [134,135]; HCC-focused validation is ongoing. Elevated kynurenine activates the aryl hydrocarbon receptor (AhR) on MDSCs and tumor cells, driving sustained MDSC expansion, Treg differentiation, and direct T-cell anergy [124]. This pathway is particularly active in HCC patients with dysbiotic microbiomes enriched for kynurenine-producing species. These findings illuminate a sophisticated “microbiota–metabolite–MDSC” interactome that operates in parallel with canonical cytokine and chemokine networks [136]. Therapeutic strategies that selectively target deleterious metabolites (e.g., engineered bacteria that degrade kynurenine or secondary bile acids) or replenish beneficial ones (e.g., next-generation butyrate-producing consortia tailored for the tumor context) are now in active preclinical development. When integrated with immune checkpoint blockade (ICB) and conventional MDSC-depleting agents, such precision microbiome–metabolite interventions hold considerable potential to dismantle systemic immunosuppression and help convert immunologically cold HCCs into more responsive tumors. Ongoing trials include NCT05690048 (FLORA: FMT with Atezolizumab in HCC) [137] NCT05750030 (FAB-HCC: FMT with Atezolizumab-Bevacizumab) NCT05032014 (Probiotic with anti-PD-1 in HCC), aiming to assess safety and efficacy in converting non-responders [120].

## 5. Future Directions and Concluding Remarks

The convergence of high-throughput sequencing, multi-omics integration, and artificial intelligence has transformed our understanding of MDSC heterogeneity and their central role in HCC immune evasion [124]. Emerging evidence consistently positions specific microbial signatures—particularly Akkermansia muciniphila and Lachnoclostridium enrichment—as robust predictors of ICB response, while dysbiosis-driven metabolite circuits (kynurenine–AhR, secondary bile acids) sustain MDSC dominance [118,119,124]. Fecal microbiota transplantation from ICB responders has already demonstrated proof-of-concept reversal of primary resistance in solid tumors, including HCC cases [138]. Multiple prospective trials (e.g., FAB-HCC, NCT05750030) have tested whether FMT can convert atezolizumab–bevacizumab non-responders into durable responders by depleting pathogenic MDSC-promoting taxa and restoring protective commensals, with preliminary results indicating feasibility and potential efficacy. Looking ahead, the most promising regimens will likely integrate microbiome engineering with multi-target MDSC blockade. Rational combinations of engineered probiotics, next-generation FMT, STAT3/CXCR4 inhibitors, and metabolic modulators (e.g., tadalafil, SQLE inhibitors) promise to dismantle the redundant immunosuppressive networks that currently limit cure rates. Artificial intelligence is accelerating this paradigm shift: deep-learning frameworks now stratify HCC patients into MDSC-high/ICB-low subtypes, identify novel druggable nodes within the MDSC–metabolite interactome (e.g., using machine learning on single-cell RNA-seq data [13,16,139]) and guide personalized microbial consortia design [139,140,141,142]. When coupled with prospective biomarker panels that incorporate baseline MDSC frequency, microbiome composition, and circulating metabolite profiles, these AI-driven strategies will enable true precision immunotherapy [141,142,143,144]. In conclusion, myeloid-derived suppressor cells represent the single most versatile and clinically actionable barrier to successful HCC immunotherapy [145]. Over the past five years, we have moved from recognizing their dominance—including increased MDSC expression in HBV-related HCC and post-locoregional therapies such as Y-90 radioembolization—to deploying multiple validated strategies—vascular normalization, locoregional priming, transcriptional reprogramming, hematopoietic suppression, and microbiome restoration—that directly neutralize MDSC-driven resistance [146,147]. The landmark successes of triplet regimens (TACE–anti-angiogenic–ICB) and emerging microbial therapeutics herald a new era in which durable cures, rather than transient responses, become achievable for a substantial proportion of patients, building on systemic therapy advancements like lenvatinib–pembrolizumab, nivolumab monotherapy, and broader updates in HCC immunotherapy [148,149,150,151].

## 6. Conclusions

In summary, myeloid-derived suppressor cells (MDSCs) represent a pivotal immunosuppressive element in the hepatocellular carcinoma (HCC) tumor microenvironment, driving resistance to immune checkpoint blockade (ICB) and contributing to poor prognosis. This review has delineated the multifaceted mechanisms underpinning MDSC function, including key signaling pathways, metabolic reprogramming, and epigenetic modifications, which collectively orchestrate immune evasion. Emerging therapeutic strategies, such as STAT3 inhibitors, metabolic modulators, epigenetic agents, microbiome interventions, and combinatorial approaches with ICB or locoregional therapies, offer promising avenues to deplete MDSCs, reprogram the TME, and enhance antitumor immunity. Clinical trials and preclinical data underscore the potential of these interventions to improve response rates, particularly in ICB-refractory patients, highlighting MDSCs as a cornerstone target for precision immunotherapy in HCC.

Looking forward, integrating artificial intelligence with multi-omics analyses will accelerate biomarker discovery for patient stratification and personalized regimens, addressing MDSC redundancies and overcoming therapeutic resistance. While challenges such as hepatotoxicity and off-target effects persist, multidisciplinary efforts combining microbial modulation, nanomedicine, and advanced imaging hold the potential to transform HCC management, ultimately extending survival and achieving durable remissions in a broader patient population.

## Figures and Tables

**Table 1:** Heterogeneity and clinical relevance of major MDSC subsets in hepatocellular carcinoma (HCC).

MDSC Subset	Key Markers (Human)	Key Markers (Mouse)	Primary Suppressive Mediators	Major Location and Distribution	Prognostic Links and Clinical Relevance in HCC
PMN-MDSC (Granulocytic)	CD11b^+^ CD14^−^ CD15^+^ (or CD66b^+^) HLA-DR^−^/low CD33	CD11b^+^ Ly6G^+^ Ly6C	High ROS, PNT, Arg-1, S100A8/A9, LOX-1	Peripheral blood (70–90% of total MDSCs); tumor tissue	Most abundant subtype; strongly correlates with advanced stage, poor OS, chemotherapy/ICB resistance [4,13,22]
M-MDSC (Monocytic)	CD11b^+^ CD14^+^ CD15^−^ HLA-DR^−^/low CD33^+^	CD11b^+^ Ly6G^−^ Ly6C	Arg-1, iNOS/NO, TGF-β, IL-10, PGE2; PD-L1; Treg induction; differentiation into TAMs	Peripheral blood; tumor tissue; lymph nodes	Higher per-cell suppressive potency; linked to post-LT recurrence, TAM differentiation, poor prognosis [2,3,5,23,31]
eMDSC (Early stage)	Lin^−^ (CD3/14/15/19/56) HLA-DR^−^ CD33^+^ CD11b^+^/low	Not well-defined (immature progenitors)	Arg-1, ROS, TGF-β (maturation-dependent)	Primarily peripheral blood of cancer patients; rare in healthy individuals	Enriched in early-stage cancers; variable role in HCC progression and some autoimmune overlap [3,10,18]
Fibrocytic MDSC (Emerging/F-MDSC)	CD11b^+^ CD33^+^ HLA-DR^−^; Fibrocyte markers (Collagen I^+^, CD34^+^, CD45^+^)	Limited data; fibrocyte-like (CD11b^+^ Collagen I^+^)	Potent TGF-β production; ECM remodeling; fibrosis and angiogenesis promotion	Tumor stroma; sites of chronic inflammation	Highly enriched in fibrotic HCC (viral/NAFLD-associated); drives fibrosis-carcinogenesis axis; associated with aggressive fibrotic tumors and poor outcome [20,21,27]

**Table 2:** Therapeutic agents targeting MDSC-mediated immunosuppression in hepatocellular carcinoma (HCC).

Drug Name	Nature/Type	Role/Mechanism in HCC Context (MDSC-Related)	Clinical Development Status (as of March 2026)
Danvatirsen (AZD9150)	STAT3 antisense oligonucleotide	Depletes STAT3 mRNA, promotes MDSC apoptosis and differentiation, reduces immunosuppression; combined with ICB	Pan-tumor + HCC expansion; Phase Ib/II (NCT03421353); limited HCC-specific cohort; ORR 26% in solid tumors with MDSC depletion; acceptable tolerability; HCC expansion ongoing
Napabucasin (BBI-608)	Small-molecule STAT3 inhibitor	Inhibits STAT3-driven fatty acid oxidation, alleviates MDSC/TAM-mediated T-cell exhaustion; synergizes with anti-PD-1	Pan-tumor (HCC preclinical); Phase II/III (failed in CRC); no active dedicated HCC trial; preclinical survival benefit in HCC models
TTI-101	Oral STAT3 inhibitor	Inhibits STAT3 signaling, impairs tumor proliferation and MDSC expansion; combined with CXCR4 antagonists or ICB	HCC-specific (advanced/refractory); Phase I completed + Ib/II ongoing (NCT03195699; NCT05440708); n = 17 HCC in Phase I (ORR 18%, CBR 54%); favorable safety, no major hematologic DLT; topline Phase II data expected H1 2026
Pexidartinib	CSF1R inhibitor	Depletes CSF1-dependent myeloid populations, reduces MDSC infiltration; synergizes with ICB	Pan-tumor (HCC exploratory); Approved for TGCT; limited HCC data; exploratory in HCC
LY3022855	CSF1R inhibitor	Similar to pexidartinib; reduces MDSC/TAM survival in TME; combined with anti-PD-L1	Pan-tumor; Phase I (NCT02718911); limited HCC cohort; safety + MDSC reduction; liver enzyme elevation noted
Tadalafil	PDE5 inhibitor (metabolic modulator)	Suppresses Arg-1, iNOS, and ROS in MDSCs; elevates cGMP; synergizes with anti-PD-1	Pan-tumor (HCC preclinical); Repurposed (Phase II in other cancers); strong preclinical synergy in HCC; no dedicated HCC trial
Terbinafine	SQLE inhibitor (antifungal repurposed)	Inhibits cholesterol biosynthesis in MDSCs, reduces infiltration; restores anti-PD-1 efficacy in MASH-HCC	Preclinical HCC only; No dedicated HCC trial; preclinical restoration of anti-PD-1 sensitivity; rare idiosyncratic hepatotoxicity
CXD101	HDAC inhibitor	Triggers tumor-cell pyroptosis via STAT1-GSDME axis, reduces MDSC infiltration; combined with PD-1 blockade	HCC-specific (post A + T); Phase II (NCT05873244); recruiting; ORR/PFS with anti-PD-1; HDAC-class AEs (fatigue, GI, hematologic)
PCI-34051	HDAC8 inhibitor	Downregulates MDSC function, promotes DC maturation; potentiates anti-PD-1/PD-L1	Preclinical HCC; No clinical trial in HCC
Entinostat	HDAC inhibitor	Neutralizes MDSCs, enhances antitumor effect of PD-1 inhibition; transcriptional reprogramming	Pan-tumor; Phase II/III; limited HCC data; no active dedicated HCC trial
Bevacizumab	Anti-VEGF monoclonal antibody	Normalizes vasculature, reduces MDSC frequencies and hypoxia; synergizes with anti-PD-L1 (A + T regimen)	HCC-specific; Approved (IMbrave150, NCT03434379); large Phase III; OS 19.2 vs. 13.4 mo; hypertension, bleeding
MTL-CEBPA	Small activating RNA (saRNA)	Upregulates C/EBP-α, interrupts STAT3-MDSC loop; depletes MDSCs and reprograms TME; combined with TKIs/ICB	HCC-specific; Phase I completed + Phase II (NCT04710641); n = 24 HCC evaluable in Phase I (DCR 41–54%, durable PR); Grade 3 TRAEs ~24%; Phase II completed, further combo studies ongoing
Icaritin	Flavonoid derivative (ERα36 inhibitor)	Inhibits IL-6/JAK2/STAT3 signaling, reduces MDSC generation and suppressive activity	HCC-specific; Approved in China (NMPA 2022); Phase III China data; mild, well tolerated
AMD3100 (Plerixafor)	CXCR4 antagonist	Blocks MDSC recruitment via CXCL12/CXCR4 axis; reduces infiltration and restores T-cell function	Pan-tumor (HCC exploratory); Approved for HSC mobilization; exploratory/combo use in HCC
3PO/PFK-015/PFK-158	PFKFB3 inhibitors (glycolysis modulators)	Reduce MDSC suppressive activity by inhibiting glycolysis; synergize with PD-1/PD-L1 blockade	Pan-tumor; Phase I completed; no active HCC trial
Lipofermata	FATP2 inhibitor (lipid metabolism)	Blocks arachidonic acid import, reduces PGE2 production in MDSCs; abolishes suppressive activity	Preclinical; No clinical trial
Etomoxir	FAO inhibitor (lipid metabolism)	Inhibits mitochondrial fatty acid oxidation in MDSCs; restores T-cell function	Preclinical; No clinical trial (mitochondrial toxicity)
ATRA (All-trans retinoic acid)	Differentiation promoter	Promotes MDSC differentiation into non-suppressive cells	Pan-tumor (HCC exploratory); Phase I/II in other cancers; exploratory in HCC
ICG-001	β-catenin/CBP inhibitor	Disrupts MDSC trafficking in β-catenin-mutant HCC; sensitizes “cold” tumors to anti-PD-1	Preclinical HCC; No clinical trial

## Data Availability

No new data were created or analyzed in this study. Data sharing is not applicable to this article.
